# A Novel Scheme for Quantitative Electromagnetic Inversion of Non-Cooperative Translational Targets Under Limited-Aperture Scenarios

**DOI:** 10.3390/s26082403

**Published:** 2026-04-14

**Authors:** Yitao Lin, Shilong Sun, Dahai Dai, Yuchen Wu, Bo Pang

**Affiliations:** College of Electronic Science and Technology, National University of Defense Technology, Changsha 410073, China; linyitao23@nudt.edu.cn (Y.L.); sunshilong@nudt.edu.cn (S.S.); wuyuchen@nudt.edu.cn (Y.W.); pangbo84826@nudt.edu.cn (B.P.)

**Keywords:** electromagnetic inverse scattering, translational target, limited-aperture, relative motion compensation, domain contraction, wavelength-dependent weighting

## Abstract

To achieve accurate localization and tackle the inevitable dual challenges of ill-posedness and strong nonlinearity in limited-aperture translational target inversion, this paper proposes a novel integrated scheme that synergistically combines a domain contraction (DC) strategy with wavelength-dependent weighting (WW) of multi-frequency data. The DC strategy dynamically reduces the solution space to mitigate ill-posedness and enhance stability, while the WW strategy strategically prioritizes lower-frequency data to mitigate nonlinear effects. This organically integrated approach, termed the domain-contracted wavelength-dependent weighting RMC-CC-CSI (DC-WW-RMC-CC-CSI) algorithm, enables a more robust and efficient inversion process. Simulation results demonstrate that our DC-WW scheme delivers significant improvements over the baseline RMC-CC-CSI method in imaging accuracy, convergence speed, noise robustness, and computational efficiency.

## 1. Introduction

Electromagnetic (EM) inverse scattering imaging technology reconstructs the electromagnetic scattering echoes in the “resonance region” received by the antennas into images representing the permittivity or conductivity distribution of the target object, which has been widely applied in real-world practice including medical imaging [1,2], oil reservoir exploration [3], subsurface object detection [4], concealed weapon detection [5] and remote sensing [6,7,8,9,10,11], etc.

The EM inverse scattering problem (ISP) is inherently nonlinear and ill-posed because of the involved governing equations, especially when the measuring conditions are unfavorable and diverse scattering data from different observation angles are not available. It is quite challenging to solve the inverse scattering problem in a reliable, robust and efficient calculation method [12]. Quantitative iterative inversion methods share better performance and stronger applicability than non-iterative methods in EM ISPs, including deterministic optimization methods such as the Born iterative method (BIM) [13], contrast source inversion (CSI) [14,15], the subspace-based optimization method (SOM) [16], and inversion methods that employ stochastic optimization algorithms [17,18,19]. They iteratively search for the optimal solution of the cost function by calculating the determined or stochastic optimization direction, which requires a good initial guess or prior knowledge in advance to ensure that the inversion process eventually converges to the global minimum. To obtain a better initial guess and overcome the shortcomings of traditional iterative inversion methods, hybrid methods (HMs) have been proposed [20,21,22,23]. HMs first obtain the geometry of the scatterer by means of linear non-iterative methods, and then iteratively restore the electromagnetic parameters of the scatterer or improve its geometry. Recently, with the rapid development of computer technology, intelligent electromagnetic computational methods based on deep learning have significantly enhanced the efficiency of inverse scattering imaging. These approaches can improve imaging accuracy for specific scenarios and even achieve super-resolution imaging. However, they often remain heavily dependent on the quantity and quality of training data [24]. This dependency poses a practical challenge for scenarios where acquiring extensive real-world measurement data is difficult or costly. Furthermore, the generalization capability of deep learning models, when confronted with unseen data distributions, new target geometries, or different frequency bands, has been a critical concern. While early data-driven models often showed limited generalization compared to conventional model-based iterative methods, recent advances have effectively addressed this limitation. A promising direction involves the integration of physical models directly into the network architecture. These physics-informed approaches have shown marked improvements in more robust and reliable generalization [25,26].

Despite remarkable innovations proposed by researchers to address multifaceted challenges in EM ISPs, the EM quantitative inverse scattering imaging for moving targets remains an underdeveloped filed. When the target moves within the observation coverage of antenna array, the acquired data from different measurement cycles will correlate with the spatial position distribution of the target at distinct time instants and the unknown EM parameters to be inverted will transform into time-varying quantities, significantly increasing the complexity of inverse scattering imaging. Recently, a novel electromagnetic quantitative inversion method for translationally moving targets via phase correlation registration of back-projection (BP) images is proposed [27]. Inspired by the concept of inverse synthetic aperture radar (ISAR), which relies on relative target-radar motion for high azimuth resolution, this approach first accomplishes accurate localization and registration [28,29] through back-projection images [30] of translational targets, then applies the relative motion-compensated cross-correlated CSI (RMC-CC-CSI) algorithm based on the cross-correlated CSI (CC-CSI) method [31] to achieve high-fidelity reconstruction.

As previously discussed, the ideal subjects for inverse scattering imaging are targets within the resonance region, where the target size is comparable to the incident wavelength. In contrast, BP imaging relies on a point-scattering model and is generally applied to targets in the optical region, where the target size is much larger than the wavelength. Consequently, applying the BP algorithm to multi-frame snapshot imaging of moving targets within the resonance region inevitably introduces blurring, deformation, and inaccuracies in the reconstructed target size and position. Although the inherent robustness of the BP algorithm ensures minimal variation between adjacent frames—enabling accurate relative displacement estimation via image registration—the uncertainty in absolute scale and position prevents direct use of BP results to define the inversion domain D. Moreover, in inverse problems, the distribution and number of unknown variables are determined by the grid positions and discretization within D, whose appropriate selection critically affects both imaging quality and computational efficiency. This issue is exacerbated under limited-aperture conditions, where the lack of multi-angle scattering data leads to a high ratio of the number of unknown variables to available measurement data, intensifying the ill-posedness of the problem. Additionally, for high-contrast objects, the contrast source distribution becomes more complex, further increasing the nonlinearity and ill-posedness. To address these challenges, in this paper, a practical and effective solution to the specific gap in limited-aperture quantitative inversion for moving targets is proposed.

We first introduce a domain contraction strategy to ensure the accurate positioning of D and the effective reduction of the number of unknowns during the iteration process. Then, a wavelength-dependent weighting strategy [32] that prioritizes low-frequency components through enhanced weighting coefficients in the cost function construction is integrated into the RMC-CC-CSI algorithm to improve performance in high-contrast scenarios. Combining domain contraction with wavelength-dependent weighting, a quantitative inverse scattering scheme for non-cooperative translational targets under limited-aperture conditions is finally proposed. The framework effectively leverages the computational efficiency and improved well-posedness from optimized data usage, along with the robustness offered by low-frequency stability. According to DC strategy, we obtain the initial D and the relative displacements from BP results across different observation instants. Then, the WW_*n*_-RMC-CC-CSI is conducted for iterative inversion. The inversion domain D is periodically refined and contracted using intermediate iterative results, and computations resume on the updated grid. Simulated data of transverse magnetic (TM) wave validations demonstrate that the proposed framework substantially improves the quantitative inversion capability for translational targets under limited-aperture.

## 2. Problem Statement and Translational Target Inversion

### 2.1. Problem Statement

**Figure 1:**
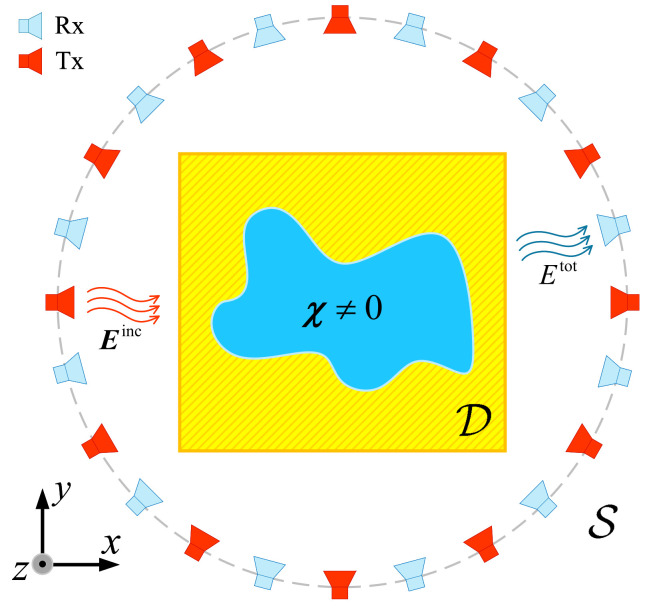
Configuration of the 2D EM ISP under TM polarization.

In the free space background, the 2D geometric configuration of the EM ISP under TM incidence is shown in Figure 1. An unknown scatterer with contrast χ lies within the imaging domain D, illuminated by TM waves with time factor of $e^{i\omega t}$, where $i=\sqrt{-1}$ represents the imaginary unit and ω is the angular frequency. S represents the measurement domain of the field. The transmitters are denoted by the subscript p∈{1,2,3…,P}, and the receivers are denoted by the subscript q∈{1,2,3,…,Q}. For the incident wave generated by each transmitter, the scattered field excited by the target is measured by the receivers. the scattering field data that contains information about the target can be used to invert the target characteristic parameters. In this paper, a vector with three components will be represented by a bold symbol. The data equation and state equation based on the finite-difference frequency-domain (FDFD) [33] are

(1) $$f_{p}=M^{S}A^{-1}\omega^{2}\chi ⊙e_{p}^{\mathrm{tot}},\;\;f_{p}\in S$$

(2) $$e_{p}^{\mathrm{tot}}=e_{p}^{\mathrm{inc}}+M^{D}A^{-1}\omega^{2}\chi ⊙e_{p}^{\mathrm{tot}},\;\;e_{p}^{\mathrm{tot}}\in D$$

where $f_{p}$ is the measurement data of the scattered field and A is the stiffness matrix under FDFD scheme. $M^{S}$ is a sampling matrix that extracts the measured data at the positions of receivers from domain S; $M^{D}$ is a mask matrix that only extracts the values defined in D, which is uniformly discretized into N×N grids of equal area. $e_{p}^{\mathrm{tot}}$ and $e_{p}^{\mathrm{inc}}$ represent the electric total field and incident field. The contrast function χ of the target satisfies $\chi =\varepsilon -\varepsilon_{b}$; here, ε is the complex permittivity of target while $\varepsilon_{b}=\epsilon_{b}-i\sigma_{b}/\omega$ is the complex permittivity of the imaging background. $\epsilon_{b}$ and $\sigma_{b}$ are the permittivity and conductivity of the background, respectively. $j_{p}=\chi ⊙e_{p}^{\mathrm{tot}}$ is defined as the contrast source, where ⊙ is the component multiplication operator. For the sake of brevity, $\omega^{2}$ will be written into A in the rest of this paper. The ISP is to reconstruct the contrast χ through the incomplete scattered field measurement data $f_{p}$.

### 2.2. Translational Target Inversion

This section briefly reviews the translational target inversion scheme established in [27], which serves as the necessary foundation for the proposed new method. The translational target inversion scheme consists of two components: fast positioning based on phase correlation registration of BP images, followed by iterative inversion for moving targets (RMC-CC-CSI).

#### 2.2.1. Phase Correlation Registration of BP Images

The radar system captures multiple-input multiple-output (MIMO) data in *M* scanning cycles during moving target observation, with *M* being the total number of MIMO scanning instances. By applying BP imaging to data from multiple cycles, we obtain a series of target “snapshots”. However, perspective variations and target extension will cause shape inconsistencies between successive BP images, which impedes reliable motion centroid determination and precise spatiotemporal localization. Therefore, the phase correlation registration algorithm is conducted to obtain translational shifts between consecutive frames. For 2D scenarios, let $R=[r_{1},r_{2},\ldots ,r_{M}]$ denote the target’s ideal center position matrix across *M* observation instants. $r_{m}={\left[x_{m},y_{m}\right]}^{T}$ is the ideal center position vector at the *m*-th instant, where m∈{1,2,…,M}. The spatial relative displacement matrix $R_{\Delta ,c}$ at different instants referencing to the position vector $r_{c}$ at specific instant *c* satisfies

(3) $$R_{\Delta ,c}=R-r_{c}\;$$

where c∈{1,2,…,M} is the selected central reference instant that determines the original inversion domain $D^{c}$ of the moving target. Broadcasting is applied to the calculations of matrix and vector.

#### 2.2.2. Relative Motion-Compensated CC-CSI Based on FDFD

During target motion, all elements defined over the discretized imaging domain D become coupled with the target’s dynamic positions. On the basis of [27], when dealing with monofrequency data, the data residual, state residual and cross-correlated residual suitable for ISP of moving targets are defined as

(4) $${\tilde{\rho }}_{m,p}^{c}=f_{m,p}-{\tilde{\Phi }}_{m}^{c}j_{m,p}^{c}\;$$

(5) $${\tilde{\gamma }}_{m,p}^{c}=\chi ⊙e_{m,p}^{\mathrm{inc},c}+\chi ⊙A^{-1}j_{m,p}^{c}-j_{m,p}^{c}$$

(6) $${\tilde{\xi }}_{m,p}^{c}=f_{m,p}-{\tilde{\Phi }}_{m}^{c}\left(\chi ⊙e_{m,p}^{\mathrm{inc},c}+\chi ⊙A^{-1}j_{m,p}^{c}\right)$$

where $f_{m,p}$ denotes the scattered field acquired through multiple MIMO scanning cycles, and $j_{m,p}^{c}$ and $e_{m,p}^{\mathrm{inc},c}$ are distributed in $D^{c}$. The motion sensing matrix is constructed as ${\tilde{\Phi }}_{m}^{c}=M_{m}^{{\tilde{S}}^{c}}A^{-1}$. ${\tilde{S}}^{c}$ is the motion-compensated relative measurement domain. $M_{m}^{{\tilde{S}}^{c}}$ denotes the motion-compensated $M^{S}$ operator referenced to central instant *c*. When M=1, above formulations reduce to a static target imaging scenario.

Through phase correlation registration of BP images, the relative position variations between target and transceivers across different observation instants can be obtained and transformed into equivalent relative transceivers’ positions vector $r_{m,q}^{c}$, which can be then used to calculate the motion sensing matrix ${\tilde{\Phi }}_{m}^{c}$. $r_{m,q}^{c}$ is formulated as

(7) $$r_{m,q}^{c}=r_{c,q}^{c}-R_{\Delta ,c}\left(:,m\right)$$

Here, $r_{c,q}^{c}$ is the known position vector of the q-th receiver in the *c*-th MIMO scanning cycle. The RMC-CC-CSI algorithm minimizes a cost function comprising the three aforementioned residuals, performing alternating updates of $j_{m,p}^{c}$ and χ during iterative optimization. The cost function for updating the contrast source $j_{m,p}^{c}$ in RMC-CC-CSI is defined as

(8) $$\begin{array}{c} C^{j^{c}}={\tilde{\eta }}^{{\tilde{S}}^{c}}\sum_{m=1}^{M}\sum_{p=1}^{P}{∥{\tilde{\rho }}_{m,p}^{c}∥}_{{\tilde{S}}^{c}}^{2}+{\tilde{\eta }}^{D^{c}}\sum_{m=1}^{M}\sum_{p=1}^{P}{∥{\tilde{\gamma }}_{m,p}^{c}∥}_{D^{c}}^{2}+{\tilde{\eta }}^{{\tilde{S}}^{c}}\sum_{m=1}^{M}\sum_{p=1}^{P}{∥{\tilde{\xi }}_{m,p}^{c}∥}_{{\tilde{S}}^{c}}^{2} \end{array}\;$$

Here, ${∥\cdot ∥}_{{\tilde{S}}^{c}}$ and ${∥\cdot ∥}_{D^{c}}$ represent the 2-norms on the relative measurement space $L^{2}\left({\tilde{S}}^{c}\right)$ and the imaging field space $L^{2}\left(D^{c}\right)$. ${\tilde{\eta }}^{{\tilde{S}}^{c}}$ and ${\tilde{\eta }}^{D^{c}}$ are formulated as

(9) $${\tilde{\eta }}^{{\tilde{S}}^{c}}=1/\left(\sum_{m=1}^{M}\sum_{p=1}^{P}{∥f_{m,p}∥}_{{\tilde{S}}^{c}}^{2}\right),\;{\tilde{\eta }}^{D^{c}}=1/\left(\sum_{m=1}^{M}\sum_{p=1}^{P}{∥\chi ⊙e_{m,p}^{\mathrm{inc},c}∥}_{D^{c}}^{2}\right)$$

Similarly, the cost function for updating χ is defined as

(10) $$C^{\chi }={\tilde{\eta }}^{D^{c}}\sum_{m=1}^{M}\sum_{p=1}^{P}{∥{\tilde{\gamma }}_{m,p}^{c}∥}_{D^{c}}^{2}+{\tilde{\eta }}^{{\tilde{S}}^{c}}\sum_{m=1}^{M}\sum_{p=1}^{P}{∥{\tilde{\xi }}_{m,p}^{c}∥}_{{\tilde{S}}^{c}}^{2}$$

Detailed implementation for multi-frequency RMC-CC-CSI is given in [27]. More information about initialization procedures and termination strategies are comprehensively described in [31].

## 3. Synthetic Data and the DC-WW_*n*_ Inversion Method

Section 2.2 concisely summarize the translational target inversion scheme from prior work, upon which our novel method is built. This section introduces the forward simulation datasets and presents the core methodological innovations we propose.

### 3.1. Forward Simulation and Measurement Configuration

Two different synthetic datasets named CrossRect profile and SqTwoDisc profile under TM polarization are efficiently generated using the finite-difference time-domain (FDTD) forward solver gprMax [34]. The hybrid solver strategy of FDTD-generated FDFD-inverted was chosen considering both computational efficiency and the need for robust algorithm testing. The systematic error it introduces by the solver mismatch is minor and manageable. The performance improvement offered by our algorithm is an inherent advantage of the method itself and therefore does not depend on a specific forward-inverse solver pair.

**Figure 2:**
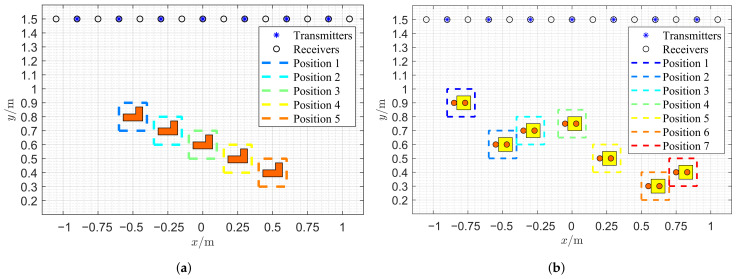
The geometry of the other profiles and their synthetic measurement configurations. (**a**) CrossRect configuration. (**b**) SqTwoDisc configuration.

**Figure 3:**
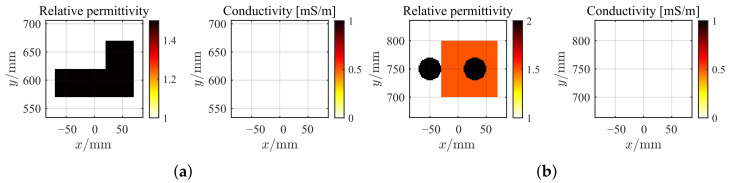
Ground truths of the datasets. (**a**) CrossRect profile at m=3. (**b**) SqTwoDisc profile at m=4.

The geometry of the these two profiles and their synthetic measurement configurations are shown in Figure 2. By comparing Figure 2a,b, the SqTwoDisc target exhibits greater complexity than the CrossRect target in both motion trajectory patterns and structural composition. The ideal center coordinates of the CrossRect target and the SqTwoDisc target at each observation instant are given in Table 1. We collected M=5 and M=7 rounds of observation instances for them separately. Taking position 3 (m=3) and position 4 (m=4) as examples, we present the ground truth images of the CrossRect profile and the SqTwoDisc profile in Figure 3. The SqTwoDisc is formed by two orthogonally intersecting rectangular components with identical relative permittivity and conductivity ($\varepsilon_{r}$ = 1.5, σ = 0 mS/m). Two rectangles share equal width of 0.5 m but different length of 1.4 m and 1 m, as shown in Figure 3a. The SqTwoDisc shown in Figure 3b consists of a square with side length of 0.1 m ($\varepsilon_{r1}$ = 1.5, σ = 0 mS/m) and two discs with the same radius of 0.02 m ($\varepsilon_{r2}$ = 2, σ = 0 mS/m). For both CrossRect and SqTwoDisc, we deploy a total of 7 transmitters and 15 receivers evenly distributed on a straight line of *y* = 1.5 m, with different spacing between the transmitter and the receiver, which is set as 0.3 m and 0.15 m. The frequency range is from 1 to 6 GHz with a step size of 0.05 GHz.

### 3.2. RMC-CC-CSI with Wavelength-Dependent Weighting

The utilization of a multi-frequency strategy can mitigate the nonlinear effects and ill-posedness of the inverse problems. This improvement will show particularly pronounced effects for high-contrast targets. Considering the inversion of multi-frequency data, the subscript *i* is used to represent the *i*-th frequency, and $i\in \{1,2,3\ldots ,N_{f}\}$, where $N_{f}$ is the total number of discrete frequencies. The residuals suitable for multi-frequency inverse scattering problem of moving targets are defined as

(11) $${\tilde{\rho }}_{m,p,i}^{c}=f_{m,p,i}-{\tilde{\Phi }}_{m,i}^{c}j_{m,p,i}^{c}$$

(12) $${\tilde{\gamma }}_{m,p,i}^{c}=\chi_{i}⊙e_{m,p,i}^{\mathrm{inc},c}+\chi_{i}⊙{A_{i}}^{-1}j_{m,p,i}^{c}-j_{m,p,i}^{c}$$

(13) $${\tilde{\xi }}_{m,p,i}^{c}=f_{m,p,i}-{\tilde{\Phi }}_{m,i}^{c}\left(\chi_{i}⊙e_{m,p,i}^{\mathrm{inc},c}+\chi_{i}⊙{A_{i}}^{-1}j_{m,p,i}^{c}\right)$$

Assuming the wavelengths of these operational frequencies satisfy $\lambda_{1}>\lambda_{2}>\cdots >\lambda_{N_{f}}$, by referring to [32], the frequency-dependent weighting coefficients $w_{i}$ are calculated by

(14) $$w_{i}={\left(\lambda_{i}/\lambda_{N_{f}}\right)}^{n}$$

where n≥0 represents an exponential parameter to be determined. According to numerical experiments conducted in [32], the optimal value of *n* is chosen as n=4. In this work, we adopt the choice of n=4 as well. The advantage and applicability of such setting under current configuration will be further discussed by subsequent numerical tests in Section 4.2.1. The fundamental principle remains that longer wavelengths receive higher weighting coefficients in the cost function. Accordingly, the modified cost functionals for contrast source and contrast updates in RMC-CC-CSI framework become

(15) $$C_{w}^{j^{c}}=\sum_{i=1}^{N_{f}}\left(w_{i}{\tilde{\eta }}_{i}^{{\tilde{S}}^{c}}\sum_{m=1}^{M}\sum_{p=1}^{P}{∥{\tilde{\rho }}_{m,p,i}^{c}∥}_{S}^{2}+w_{i}{\tilde{\eta }}_{i}^{D^{c}}\sum_{m=1}^{M}\sum_{p=1}^{P}{∥{\tilde{\gamma }}_{m,p,i}^{c}∥}_{D^{c}}^{2}+w_{i}{\tilde{\eta }}_{i}^{{\tilde{S}}^{c}}\sum_{m=1}^{M}\sum_{p=1}^{P}{∥{\tilde{\xi }}_{m,p,i}^{c}∥}_{{\tilde{S}}^{c}}^{2}\right)$$

(16) $$C_{w}^{\chi }=\sum_{i=1}^{N_{f}}\left(w_{i}{\tilde{\eta }}_{i}^{D^{c}}\sum_{m=1}^{M}\sum_{p=1}^{P}{∥{\tilde{\gamma }}_{m,p,i}^{c}∥}_{D^{c}}^{2}+w_{i}{\tilde{\eta }}_{i}^{{\tilde{S}}^{c}}\sum_{m=1}^{M}\sum_{p=1}^{P}{∥{\tilde{\xi }}_{m,p,i}^{c}∥}_{{\tilde{S}}^{c}}^{2}\right)$$

When n=0, wavelength-dependent weighted RMC-CC-CSI (WW_*n*_-RMC-CC-CSI) reduces to conventional RMC-CC-CSI.

### 3.3. Domain Contraction Strategy

This section details the proposed domain contraction strategy. The process begins with initializing the region of interest (ROI) by applying a designed expansion to the original target area detected via BP imaging, then a contraction procedure during iterative inversion is applied.

#### 3.3.1. Inversion Domain Initialization with Expansion

In order to accurately reconstruct the scatterers, a multi-view, multi-base and multi-frequency strategy is required. However, in the case of non-cooperative moving target inversion, the implementation is often constrained to a limited-aperture MIMO array. This limitation, combined with the mismatch with frequencies in the resonance region, degrades the accuracy of the BP imaging results, therefore affecting the localization of inversion domain $D^{c}$ determined by BP image at the selected reference instant *c*.

**Figure 4:**
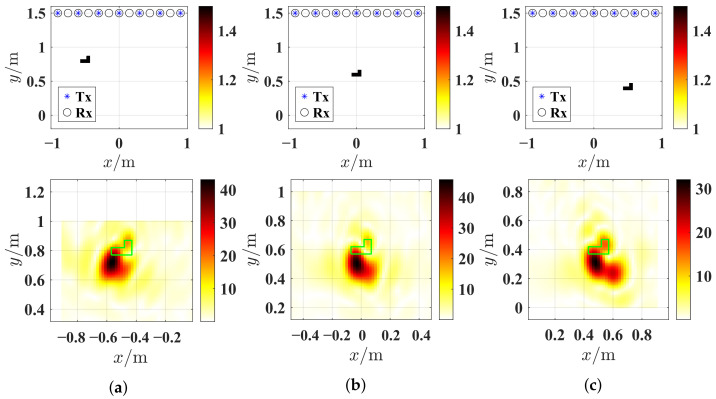
The measurement configurations and BP results of CrossRect obtained at different cycles of MIMO observation instants. The frequencies are 1–2 GHz with 0.05 GHz step. (**a**) Scanning cycle 1. (**b**) Scanning cycle 3. (**c**) Scanning cycle 5. The ground truth area is drawn with a green line.

Let the multi-frame BP results obtained from various time instants be designated as $I_{\mathrm{BP}}$. Extensive experimental results based on MIMO linear arrays have revealed that when BP imaging is performed with limited-view and resonance-frequency data, the electromagnetic waves penetrate from the air medium into the target with different dielectric constants, resulting in a reduction in propagation velocity. The received echoes therefore exhibit a time delay under the assumption of free-space propagation at the speed of light, which causes the target’s position in $I_{\mathrm{BP}}$ to exhibit a “shift” along the line connecting the phase center of the MIMO equivalent virtual array and the target’s reference center, shifting backward from its true location plotted in the green line, as illustrated in Figure 4.

However, the backward deviation in BP imaging relative to the ideal result can currently only be qualitatively identified in direction, while a quantitative estimation of its exact magnitude remains challenging. This is primarily attributed to three indeterminate factors: first, according to $v=\frac{1}{\sqrt{\epsilon \mu }}$, the wave propagation velocity *v* within the target medium depends on its electromagnetic parameters, and the unknown internal properties of the target prevent the determination of velocity variations; second, the actual target-to-array distance varies with different observation geometries and target trajectories; and third, the complex multiple scattering effects in the resonance regime hinder direct echo modeling or estimation. Consequently, the indeterminacy in echo time delays ultimately prevents precise determination of the BP shift.

Leveraging this prior knowledge, the first step of domain contraction strategy lies not in the precise initialization of ROI, but in ensuring the actual target region is fully enclosed. We utilize an Otsu thresholding method [35] to extract the positional information of the “ghost target” in the BP result $I_{\mathrm{BP}}^{c}$ at reference instant *c* as ${\mathrm{ROI}}_{\left(0\right)}$:

(17) $${\mathrm{ROI}}_{\left(0\right)}=\left[x_{\left(0\right)}^{\min},y_{\left(0\right)}^{\min},x_{\left(0\right)}^{\max},y_{\left(0\right)}^{\max}\right]$$

where $x_{\left(0\right)}^{\min},x_{\left(0\right)}^{\max}$ and $y_{\left(0\right)}^{\min},y_{\left(0\right)}^{\max}$ represent the minimum or maximum position coordinates of the original grid in the *x*, *y* directions, respectively. After the original localization process, the region of ${\mathrm{ROI}}_{\left(0\right)}$ has a grid dimension of $N_{x}^{\left(0\right)}\times N_{y}^{\left(0\right)}$ and a physical size of $L_{x}^{\left(0\right)}\times L_{y}^{\left(0\right)}$, where $N_{x}^{\left(0\right)}$ and $N_{y}^{\left(0\right)}$ represent the total number of grids, and $L_{x}^{\left(0\right)}=x_{\left(0\right)}^{\max}-x_{\left(0\right)}^{\min}$ and $L_{y}^{\left(0\right)}=y_{\left(0\right)}^{\max}-y_{\left(0\right)}^{\min}$ the side lengths along two directions. The original target reference center is as follows:

(18) $$\left(x_{\left(0\right)}^{\mathrm{ref}},y_{\left(0\right)}^{\mathrm{ref}}\right)=\left(\frac{x_{\left(0\right)}^{\min}+x_{\left(0\right)}^{\max}}{2},\frac{y_{\left(0\right)}^{\min}+y_{\left(0\right)}^{\max}}{2}\right)$$

The key contribution of our work lies in the DC-WW_*n*_ inversion strategy conducted after ensuring the actual target region is fully enclosed. To enhance engineering applicability, ${\mathrm{ROI}}_{\left(0\right)}$ is extended along the direction opposite to the drift observed in the BP imaging as a preprocessing measure. The extent of this expansion is determined by the ambiguous target scale $L_{s}=\max\left\{L_{x}^{\left(0\right)},L_{y}^{\left(0\right)}\right\}$ detected in the original localization step. Since the BP shift is unable to be determined when confronted with a non-cooperative target, it is our priority to ensure that the target is encompassed within the inversion domain through expansion, rather than developing a precisely designed expansion standard for any possible situations. Under our simulated scenarios in this work, we set $s=L_{s}/2$, which is the half scale. If more conservative measures are taken, a larger expansion ratio relative to the target scale can be selected. This modified and expanded ${\mathrm{ROI}}_{\left(0\right)}$ is termed ${\mathrm{ROI}}_{\left(1\right)}$, which is utilized as the initial domain for the subsequent iterative inversion:

(19) $$\begin{array}{c} {\mathrm{ROI}}_{\left(1\right)}=\left[x_{\left(1\right)}^{\min},y_{\left(1\right)}^{\min},x_{\left(1\right)}^{\max},y_{\left(1\right)}^{\max}\right]=\left[x_{\left(1\right)}^{\mathrm{ref}}-\frac{L_{x}^{\left(1\right)}}{2},y_{\left(1\right)}^{\mathrm{ref}}-\frac{L_{y}^{\left(1\right)}}{2},x_{\left(1\right)}^{\mathrm{ref}}+\frac{L_{x}^{\left(1\right)}}{2},y_{\left(1\right)}^{\mathrm{ref}}+\frac{L_{y}^{\left(1\right)}}{2}\right] \end{array}$$

Here, the modified target reference center is

(20) $$\left(x_{\left(1\right)}^{\mathrm{ref}},y_{\left(1\right)}^{\mathrm{ref}}\right)=\left(x_{\left(0\right)}^{\mathrm{ref}},y_{\left(0\right)}^{\mathrm{ref}}\right)-\left(\frac{1}{2}s\cos\theta ,\frac{1}{2}s\sin\theta \right)$$

and $L_{x}^{\left(1\right)}=L_{x}^{\left(0\right)}+\left|s\cos\theta \right|$, $L_{y}^{\left(1\right)}=L_{y}^{\left(0\right)}+\left|s\sin\theta \right|$ are the side lengths of ${\mathrm{ROI}}_{\left(1\right)}$, where $\theta =arctan(\frac{y_{\left(0\right)}^{\mathrm{ref}}-Y^{\mathrm{ref}}}{x_{\left(0\right)}^{\mathrm{ref}}-X^{\mathrm{ref}}})$. In this paper, $\left(X^{\mathrm{ref}},Y^{\mathrm{ref}}\right)$ is the physical symmetric center coordinate of MIMO linear array.

#### 3.3.2. Domain Contraction During Iteration

The subsequent WW_*n*_-RMC-CC-CSI iterative optimization is initialized on the inversion domain $D^{c}=\mathrm{ROI}\left(1\right)$. Due to the strong multiple scattering effect in BP imaging, the results often exceed the range of the real target area. While ensuring the inclusion of the true target location in the previous domain initialization, ${\mathrm{ROI}}_{\left(1\right)}$ inevitably contains redundant non-target grids, which complicates the global solution search and increases the risk of converging to local minima, especially under the challenge of limited-aperture data. To progressively refine $D^{c}$ towards a more precise target location, the idea of further contraction during the process of iterative inversion naturally arises.

Let $\ell_{DC}$ denote the contraction interval. Once the iteration count reaches $\ell_{DC}$, the Otsu thresholding method is operated again to extract the boundaries of a new, contracted domain ${\mathrm{ROI}}_{\left(2\right)}$. All variables previously defined on ${\mathrm{ROI}}_{\left(1\right)}$ are then updated to this refined ${\mathrm{ROI}}_{\left(2\right)}$ for subsequent iterations. This strategy reduces the ratio between the unknown and the available observation data, the search domain of the solution can be limited and some local minima can be skipped. The associated time consumption and ill-posedness, which scale with the number of unknowns, are alleviated after domain contraction. Consequently, the DC-WW_*n*_ inversion framework is proposed. Algorithm 1 shows the details of domain-contracted wavelength-dependent weighting RMC-CC-CSI (DC-WW_*n*_-RMC-CC-CSI).

In practice, the domain contraction parameter $\ell_{DC}$ is chosen to balance contour refinement efficacy and computational efficiency based on subsequent numerical experiments and analysis. The selection principle of $\ell_{DC}$ will be discussed further in Section 4.2.2. Here, the required total number of iterations $\ell_{\max}$ depends on the target’s contrast, geometric configuration, and the noise level in the data (data with low signal-to-noise ratios are prone to premature overfitting). In practical applications, a feasible approach is to empirically estimate $\ell_{\max}$ through preliminary trials involving inversions of similar targets [32,36].
**Algorithm 1:** Multi-frequency DC-WW_*n*_-RMC-CC-CSI
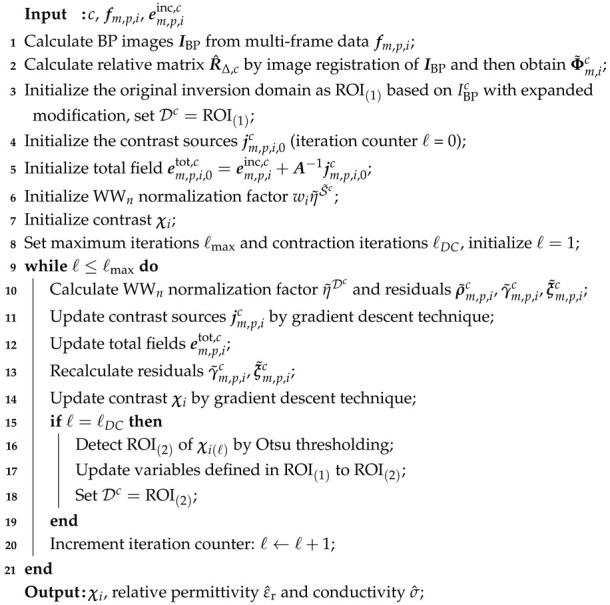


## 4. Inversion Results

In this section, basic procedures of DC-WW_*n*_-RMC-CC-CSI are shown and discussed through the inversion of the CrossRect profile. Numerical experiments and comparisons are carried out and analyzed from the aspects of inversion accuracy, convergence speed, anti-noise ability and computational efficiency. The anti-noise ability is tested by adding complex Gaussian white noise to the total field. To evaluate the registration accuracy of BP imaging, we introduce the root mean square error (RMSE) that reflects the overall distribution of registration errors. The peak signal-to-noise ratio (PSNR) is used to quantitatively assess the inversion accuracy of the reconstructed scatterers. In the following Section 4, we utilize the fast inversion algorithms mentioned in [37].

### 4.1. The Basic Procedures of DC-WW_n_-RMC-CC-CSI

We utilize all five rounds of the linear array MIMO data form CrossRect dataset to show the basic procedures of DC-WW_*n*_-RMC-CC-CSI. The electromagnetic inversion for moving target is performed using the instantaneous spatial position at *c* = 2 as the reference positional center. According to [27], the selection of c∈{1,2,…,M} is arbitrary since the aperture synthesis through relative motion compensation maintains invariance under any reference instant choice when processing all MIMO data at selected observation instants. This specific choice of *c* = 2 solely enables controlled comparison through different methods at the same target position, which does not affect the performance of DC-WW_*n*_-RMC-CC-CSI.

The radar BP imaging area is set to be [−0.9, 0.9] m × [0, 1] m, discretized into 100 × 100 grid units, and employing 21 frequency points spanning 1–2 GHz with 0.05 GHz step. Each BP imaging frame requires less than 1 s for computation, revealing the targets’ different spatial distribution with good consistency. The phase correlation method was applied to register consecutive BP frames to obtain the estimated relative motion displacement matrix ${\hat{R}}_{\Delta ,2}$. Ideal relative motion displacement matrix $R_{\Delta ,2}$ are calculated according to (3) and the ideal center position matrix R of the CrossRect profile given in Table 1. Subsequently, we convert ${\hat{R}}_{\Delta ,2}$ into relative position vectors $r_{m,q}^{2}$ on the relative measurement domain ${\tilde{S}}^{2}$ and obtain the motion sensing matrix ${\tilde{\Phi }}_{m}^{2}$ to perform EM inverse scattering inversion for the moving target.

**Figure 5:**
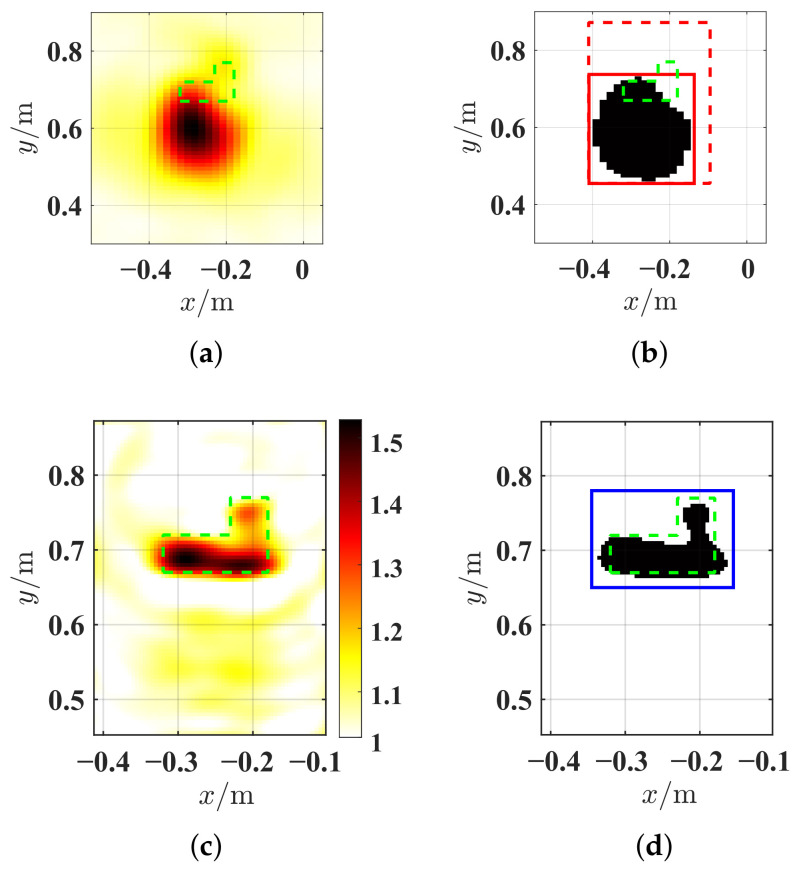

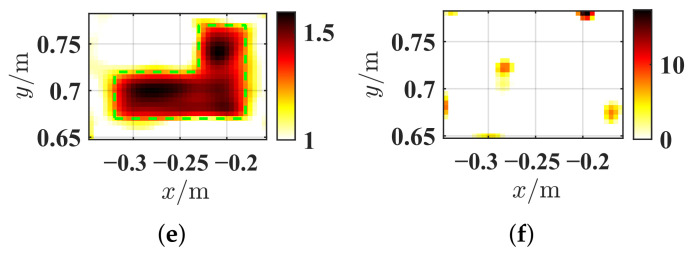
Basic procedures of DC-WW_*n*_-RMC-CC-CSI, *n* = 4. (**a**) Original BP result $I_{\mathrm{BP}}^{2}$. (**b**) Initializing the inversion domain $D^{2}$ from ${\mathrm{ROI}}_{\left(0\right)}$ to ${\mathrm{ROI}}_{\left(1\right)}$. (**c**) The reconstructed relative permittivity after $\ell_{DC}=500$ iterations. (**d**) Detecting constructed domain ${\mathrm{ROI}}_{\left(2\right)}$ during iterations. (**e**,**f**) Final reconstructed relative permittivity and conductivity [mS/m] during $5\times 10^{3}$ iterations. The true contour of the target is delineated by a green dashed line. The ${\mathrm{ROI}}_{\left(0\right)}$, ${\mathrm{ROI}}_{\left(1\right)}$ and ${\mathrm{ROI}}_{\left(2\right)}$ are delineated by a red line, red dashed line and blue line, respectively.

Based on $I_{\mathrm{BP}}^{2}$ shown in Figure 5a, the BP imaging result at *c* = 2, and by Otsu thresholding method, a rough approximation of the initial object’s border ${\mathrm{ROI}}_{\left(0\right)}$ is estimated and the 2D inversion domain $D^{2}$ was modified to ${\mathrm{ROI}}_{\left(1\right)}$ through initialization with expansion on ${\mathrm{ROI}}_{\left(0\right)}$ shown in Figure 5b. Then, the data of six frequencies selected by equal wavelength 0.05 m between 1 GHz and 6 GHz are inverted. The corresponding wavelengths are $\lambda_{1}=0.3m$, $\lambda_{2}=0.25m$, $\lambda_{3}=0.20m$, …, and $\lambda_{6}=0.05m$. This frequency selection strategy effectively exploits wavelength diversity within the resonant frequency bands of the targets while maintaining computational efficiency, which will be consistently applied in subsequent inversion tests. After $\ell_{DC}$ iterations, assuming $\ell_{DC}=500$, here, contracted domain ${\mathrm{ROI}}_{\left(2\right)}$ is also detected by the thresholding in Figure 5c,d. Final reconstructed results are given in Figure 5e,f. It is observed that the domain contraction strategy serves for the precise localization of the translational target and thus determines a proper inversion domain for the introduced wavelength-dependent weighting scheme to achieve accurate quantitative inversion for translational targets.

### 4.2. Inversion Performance

Building upon the validated effectiveness of our scheme for quantitative inversion of non-cooperative translational target in unknown scenarios, we conduct systematic comparative experiments under controlled parameters to rigorously assess DC-WW_*n*_-RMC-CC-CSI’s performance. Inversion approaches for comparative analysis are as follows:

1. DC-WW_*n*_-RMC-CC-CSI for practical implementation in non-cooperative scenarios: with estimated target positions matrix ${\hat{R}}_{\Delta ,c}$ calculated from BP images registration.
2. OPT-DC-WW_*n*_-RMC-CC-CSI: Ideal case with a priori knowledge of motion trajectories, which means that $R_{\Delta ,c}$, the true spatial relative position matrix of the moving target at each scanning cycle, is known, and optimal DC-WW_*n*_-RMC-CC-CSI can be achieved.
3. OPT-WW_*n*_-RMC-CC-CSI: Ideal case of WW_*n*_-RMC-CC-CSI, original translational moving target inversion scheme with only wavelength-dependent weighting.
4. OPT-DC-RMC-CC-CSI: Ideal case of DC-RMC-CC-CSI, original translational moving target inversion scheme with only domain contraction.
5. OPT-RMC-CC-CSI: Ideal case of RMC-CC-CSI, original translational moving target inversion scheme.

All algorithms are conducted to test the strength of the domain contraction strategy and wavelength-dependent weighting.

#### 4.2.1. The Choice of *n*

In this section, we discuss the suitable choice of the exponential parameter *n*. By adding complex Gaussian white noise with a high SNR level of 20 dB to simulated datasets, we enable robust comparative evaluation of inversion algorithms under different choices of *n* value while maintaining controlled noise contamination.

Since the DC-WW_*n*_ strategy is based on the WW_*n*_ algorithm for region contraction operations, we use the OPT-WW_*n*_-RMC-CC-CSI algorithm as the baseline to investigate the inversion effects under different parameter *n*. Under ideal cases, the motion sensing matrix ${\tilde{\Phi }}_{m,i}^{c}$ is directly calculated through the true spatial relative position matrix $R_{\Delta ,c}$. As the two examples of low contrast targets, we employ all of MIMO data for CrossRect dataset and SqTwoDisc dataset and follow the frequency selection strategy mentioned in Section 4.1, utilizing six frequencies selected by equal wavelength 0.05 m between 1 GHz and 6 GHz.

**Figure 6:**
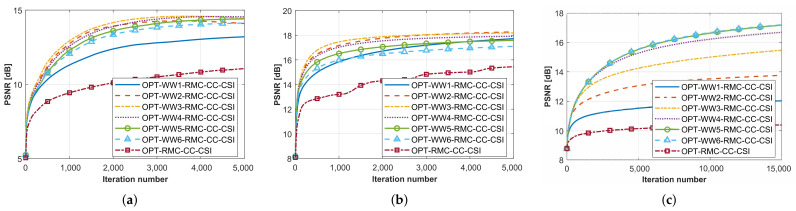
PSNR curves in terms of the iteration number when processing datasets by OPT-WW_*n*_-RMC-CC-CSI methods with different *n* values under SNR = 20 dB. (**a**) CrossRect. (**b**) SqTwoDisc. (**c**) SqTwoDisc with higher contrast.

The ideal reconstructed PSNR curves of CrossRect and SqTwoDisc using methods under different *n* values are shown in Figure 6a,b, where *n* ranges from 0 to 6 with OPT-RMC-CC-CSI representing the OPT-WW_*n*_-RMC-CC-CSI method when n=0. It is observed that the inversion results are more favorable when n=2∼4. For a target with higher contrast, we keep the conductivity unchanged and increase the relative permittivity of SqTwoDisc data to $\varepsilon_{r1}$ = 3, $\varepsilon_{r2}$ = 6. While maintaining the previous grid unit length and frequency points for inversion, the PSNR results are shown in Figure 6c. The inversion performance improves significantly with the increase in *n* before n=4. However, when *n* continues to increase beyond 4, the magnitude of performance improvement becomes increasingly marginal, with the performance remaining nearly unchanged when n=5∼6. Comparative analysis of these curves reveals an empirical optimal value of n=4, which ensures a robust inversion balance for targets spanning from low to higher contrast.

#### 4.2.2. The Selection Principles of $\ell_{DC}$

**Figure 7:**
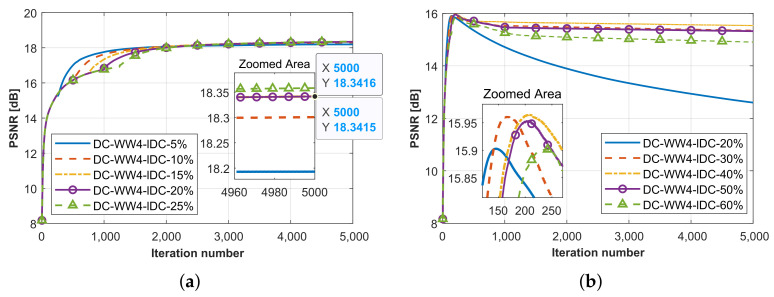
PSNR curves in terms of iteration number when processing SqTwoDisc dataset by proposed method under high and low SNR levels. (**a**) SNR = 20 dB. (**b**) SNR = 5 dB.

In this section, we further discuss the selection principle of $\ell_{DC}$. By taking the SqTwoDisc target as an example, we conducted a comparative evaluation of the proposed algorithms with different selections of the parameter $\ell_{DC}$, while maintaining controlled noise interference across varying SNR levels. Following the same frequency selection strategy, PSNR curves of SqTwoDisc using methods with different $\ell_{DC}$ values under SNR = 20 dB and 5 dB are shown in Figure 7a,b. A key observation is the algorithm’s SNR-dependent parameter selection. The suitable $\ell_{DC}$ for higher SNR favors a smaller value with $10∼25\%\;\ell_{\max}$, while a lower SNR requires a larger value ranging within $30∼50\%\;\ell_{\max}$ for better performance.

By appropriately choosing the $\ell_{DC}$, the robustness of threshold segmentation can be further ensured. Specifically, while reducing $\ell_{DC}$ improves computational efficiency, it is also essential to avoid setting the value too low. This ensures that the initial reconstruction of the target’s structural outline is achieved before proceeding with domain contraction, thereby preventing erroneous target identification and extraction. In this work, we choose $\ell_{DC}\approx 10\%\;\ell_{\max}$ under low-noise conditions (noise-free, SNR = 20 dB, 10 dB) and $\ell_{DC}\approx 40\%\;\ell_{\max}$ under stronger noise interference (SNR = 5 dB, 0 dB). In addition, the required maximum number of iterations $\ell_{\max}$ is determined using the OPT-WW_*n*_-RMC-CC-CSI algorithm as a baseline, where $\ell_{\max}$ is set to the iteration count at which the baseline algorithm reaches its optimal performance.

#### 4.2.3. Test with Multi-Frequency Synthetic Data

**Figure 8:**
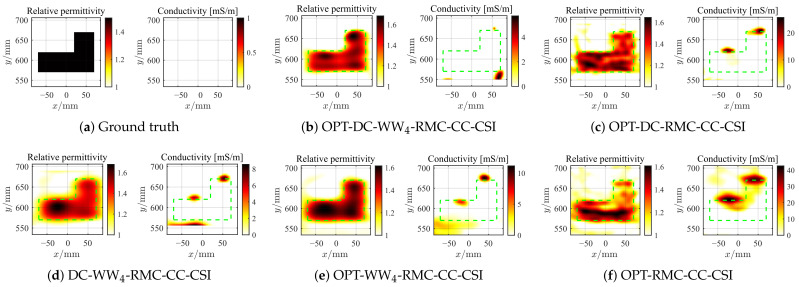
Ground truth (**a**) and inverted results of CrossRect by OPT-DC-WW_4_-RMC-CC-CSI (**b**), OPT-DC-RMC-CC-CSI (**c**), DC-WW_4_-RMC-CC-CSI (**d**), OPT-WW_4_-RMC-CC-CSI (**e**) and OPT-RMC-CC-CSI (**f**), respectively. SNR = 20 dB, $\ell_{DC}=10\%\;\ell_{\max}$, $\ell_{\max}=4390$. The true spatial contour of the target is delineated by green dashed lines.

**Figure 9:**
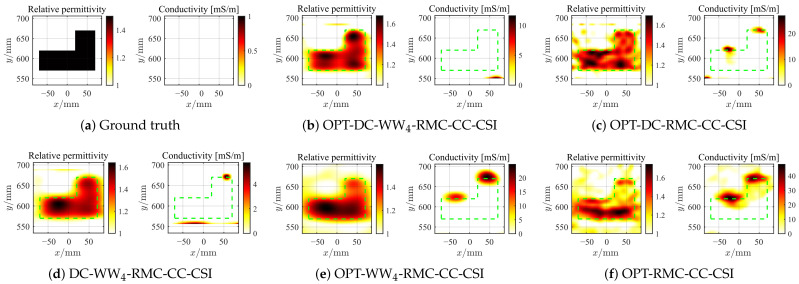
Ground truth (**a**) and inverted results of CrossRect by OPT-DC-WW_4_-RMC-CC-CSI (**b**), OPT-DC-RMC-CC-CSI (**c**), DC-WW_4_-RMC-CC-CSI (**d**), OPT-WW_4_-RMC-CC-CSI (**e**) and OPT-RMC-CC-CSI (**f**), respectively. SNR = 5 dB, $\ell_{DC}=40\%\;\ell_{\max}$, $\ell_{\max}=460$. The true spatial contour of the target is delineated by green dashed lines.

**Figure 10:**
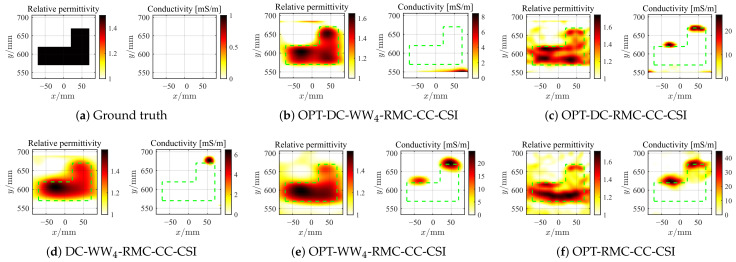
Ground truth (**a**) and inverted results of CrossRect by OPT-DC-WW_4_-RMC-CC-CSI (**b**), OPT-DC-RMC-CC-CSI (**c**), DC-WW_4_-RMC-CC-CSI (**d**), OPT-WW_4_-RMC-CC-CSI (**e**) and OPT-RMC-CC-CSI (**f**), respectively. SNR = 0 dB, $\ell_{DC}=40\%\;\ell_{\max}$, $\ell_{\max}=216$. The true spatial contour of the target is delineated by green dashed lines.

To ensure a consistent standard for effectively comparing inversion performance and calculating the PSNR across different methods, whose inversion domains may vary in size and location after domain contraction, we resample the optimal inversion results from all algorithms onto a common grid. The estimated spatial relative position matrix ${\hat{R}}_{\Delta ,c}$ of the moving target obtained through BP images registration are shown in Table 2. Compared with the ideal $R_{\Delta ,c}$ calculated by (3) and ideal R shown in Table 1, we employ RMSE to test the accuracy of registration. The mathematical formulation of RMSE is

(21) $$\mathrm{RMSE}=\sqrt{\frac{\sum_{m=1}^{M}∥r_{m,q}^{c}-{\hat{r}}_{m,q}^{c}{∥}^{2}}{M}}$$

where ${\hat{r}}_{m,q}^{c}=r_{c,q}^{c}-{\hat{R}}_{\Delta ,c}$ according to Equation (7). For both CrossRect and SqTwoDisc, the BP imaging area is set to be [−1.1, 1.1] m × [−0.2, 1.8] m, discretized into 100 × 100 grid units. As the first example, we employ all five rounds of MIMO data and BP frequencies spanning 1–2 GHz with 0.05 GHz step for the CrossRect dataset. The 2D inversion domain $D^{3}={\mathrm{ROI}}_{\left(1\right)}$ is decided based on $I_{\mathrm{BP}}^{3}$ with expanded modification and with a grid unit length of 5 mm. Similarly, by following the same frequency selection strategy, the reconstructed optimal contrast profiles of CrossRect using different methods under different levels of noise interference are shown in Figure 8, Figure 9 and Figure 10. When confronted with data perturbed by low-level noise (SNR = 20 dB), all methods achieve accurate reconstruction except OPT-RMC-CC-CSI, yielding certain deformation distortion. By adding random noise with SNR = 5 dB and 0 dB, due to the limited viewing angle provided by the linear array, the optimal inversion result of conventional OPT-RMC-CC-CSI further deteriorates.

**Figure 11:**
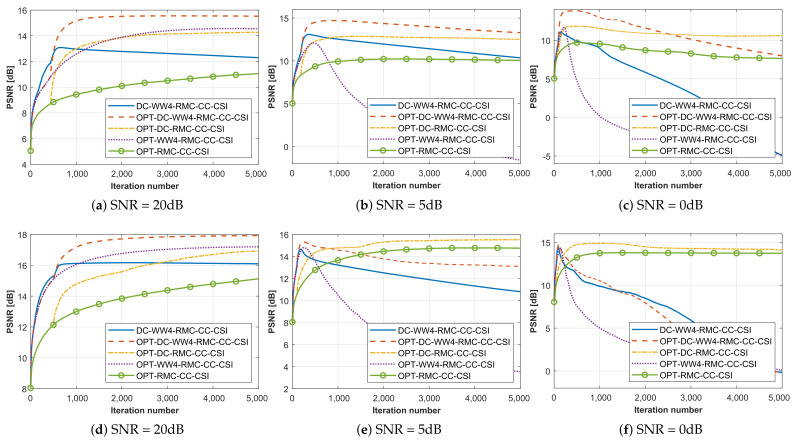
PSNR curves in terms of the iteration number when processing two datasets by different methods under situation of SNR = 20 dB, 5 dB or 0 dB. (**a**–**c**) CrossRect. (**d**–**f**) SqTwoDisc.

In addition, we compare the PSNR values in Figure 11a–c to evaluate the algorithm performance and quality, with a higher value indicating superior reconstruction precision. Among them, OPT-DC-WW_4_-RMC-CC-CSI keeps the best inversion accuracy and the fastest convergence speed, benefiting from the organic combination of DC and WW strategies. Other methods with one of the DC- or WW_4_-components also show better performance than that of the original OPT-RMC-CC-CSI algorithm at the same SNR. The low RMSE values 0.0067 and 0.0087 in Table 2 indicate that high-precision relative displacement information was obtained through BP imaging and image registration when SNR = 20 dB and 5 dB, which are close to the ideal relative coordinates of the target in Table 1. Consequently, the PSNR curve of the DC-WW_4_-RMC-CC-CSI for non-cooperative target also approaches that of the ideal OPT-DC-WW_4_-RMC-CC-CSI. However, when the SNR decreases to 0 dB, the registration accuracy of the BP image degrades (RMSE = 0.0120), leading to an increased discrepancy between the inversion results and the ideal values.

**Figure 12:**
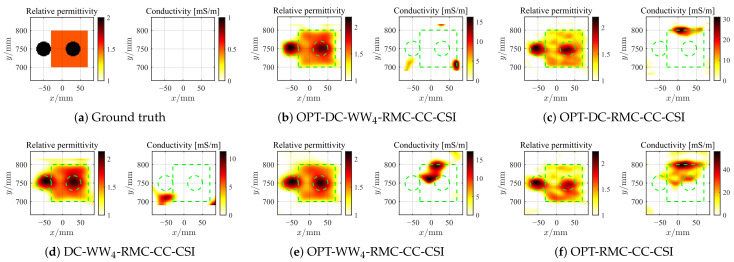
Ground truth (**a**) and inverted results of SqTwoDisc by OPT-DC-WW_4_-RMC-CC-CSI (**b**), OPT-DC-RMC-CC-CSI (**c**), DC-WW_4_-RMC-CC-CSI (**d**), OPT-WW_4_-RMC-CC-CSI (**e**) and OPT-RMC-CC-CSI (**f**), respectively. SNR = 20 dB, $\ell_{DC}=10\%\;\ell_{\max}$, $\ell_{\max}=5\times 10^{3}$. The true spatial contour of the target is delineated by green dashed lines.

**Figure 13:**
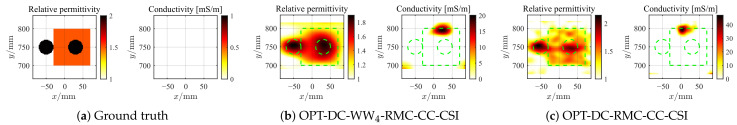

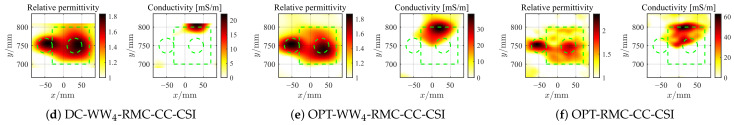
Ground truth (**a**) and inverted results of SqTwoDisc by OPT-DC-WW_4_-RMC-CC-CSI (**b**), OPT-DC-RMC-CC-CSI (**c**), DC-WW_4_-RMC-CC-CSI (**d**), OPT-WW_4_-RMC-CC-CSI (**e**) and OPT-RMC-CC-CSI (**f**), respectively. SNR = 5 dB, $\ell_{DC}=40\%\;\ell_{\max}$, $\ell_{\max}=254$. The true spatial contour of the target is delineated by green dashed lines.

**Figure 14:**
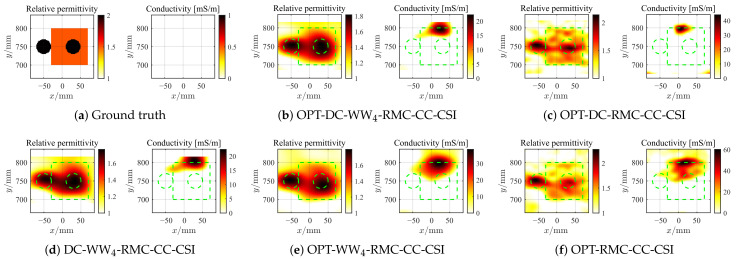
Ground truth (**a**) and inverted results of SqTwoDisc by OPT-DC-WW_4_-RMC-CC-CSI (**b**), OPT-DC-RMC-CC-CSI (**c**), DC-WW_4_-RMC-CC-CSI (**d**), OPT-WW_4_-RMC-CC-CSI (**e**) and OPT-RMC-CC-CSI (**f**), respectively. SNR = 0 dB, $\ell_{DC}=40\%\;\ell_{\max}$, $\ell_{\max}=125$. The true spatial contour of the target is delineated by green dashed lines.

As the second example, we employ all seven rounds of data for SqTwoDisc dataset. BP frequencies spanning 1–3 GHz with 0.05 GHz step involve more higher frequencies since the composition of SqTwoDisc is more complex. The 2D inversion domain $D^{4}$ is calculated by fixing *c* = 4. We also set the grid unit length to 5 mm and the same frequencies between 1 GHz and 6 GHz are inverted. The reconstructed optimal profiles of SqTwoDisc during $5\times 10^{3}$ iterations under different noise interference are shown in Figure 12, Figure 13 and Figure 14 and the PSNR curves are shown in Figure 11d–f. The advantages of OPT-DC-WW_*n*_-RMC-CC-CSI in terms of accuracy, convergence speed and noise immunity remain pronounced under noise-free conditions. As strong noise interference is introduced, the advantage of OPT-DC-WW_4_-RMC-CC-CSI over OPT-DC-RMC-CC-CSI essentially disappears and OPT-DC-WW_4_-RMC-CC-CSI exhibits overfitting at an earlier stage. In fact, by examining the previous sets of simulated results, it becomes evident that when the DC strategy is already employed, the inclusion of WW_4_ provides only marginal improvement in PSNR values. This is because, when dealing with these low-contrast targets, the inherent nonlinearity of the inverse problem is relatively weak. In such cases, excessive reliance on low-frequency information may not significantly mitigate the problem’s nonlinearity. Instead, it could reduce the utilization of richer frequency information, potentially leading the solution to a local optimum. However, in comparison, the relative permittivity distribution in the results of these WW_4_-type methods is more uniform and regular. In addition, as shown in Table 2, the presence of strong noise increases the overall registration error of DC-WW_4_-RMC-CC-CSI, which could make its result less comparable to that of OPT-DC-WW_4_-RMC-CC-CSI under ideal conditions. Nonetheless, clear target contour and positional information of the target is largely preserved.

#### 4.2.4. Further Test with Higher Contrast Target

**Figure 15:**
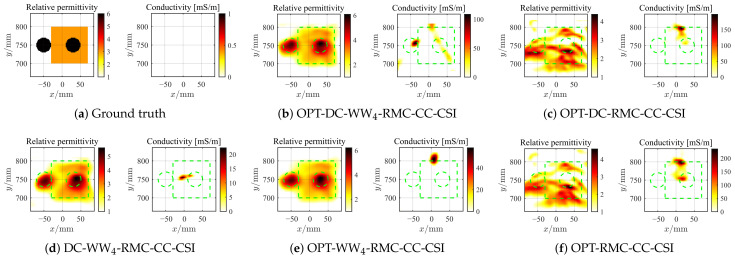
Ground truth (**a**) and inverted results of SqTwoDisc by OPT-DC-WW_4_-RMC-CC-CSI (**b**), OPT-DC-RMC-CC-CSI (**c**), DC-WW_4_-RMC-CC-CSI (**d**), OPT-WW_4_-RMC-CC-CSI (**e**) and OPT-RMC-CC-CSI (**f**), respectively. SNR = 20 dB, $\ell_{DC}=10\%\;\ell_{\max}$, $\ell_{\max}=1.5\times 10^{4}$. The true spatial contour of the target is delineated by green dashed lines.

**Figure 16:**
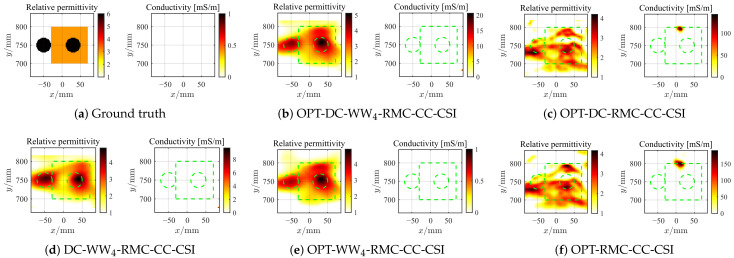
Ground truth (**a**) and inverted results of SqTwoDisc by OPT-DC-WW_4_-RMC-CC-CSI (**b**), OPT-DC-RMC-CC-CSI (**c**), DC-WW_4_-RMC-CC-CSI (**d**), OPT-WW_4_-RMC-CC-CSI (**e**) and OPT-RMC-CC-CSI (**f**), respectively. SNR = 5 dB, $\ell_{DC}=40\%\;\ell_{\max}$, $\ell_{\max}=3317$. The true spatial contour of the target is delineated by green dashed lines.

**Figure 17:**
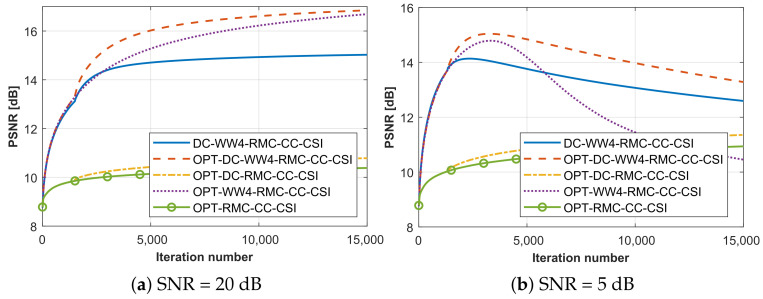
PSNR curves in terms of the iteration number when processing SqTwoDisc with higher contrast dataset by different methods under situation of SNR = 20 dB and 5 dB.

Next, we consider the SqTwoDisc target with higher contrast ($\varepsilon_{r1}$ = 3, $\varepsilon_{r2}$ = 6). While maintaining previous radar BP imaging parameters, we continue to fix the reference center position as *c* = 4 and utilize the same grid unit length and frequencies. The inversion results are shown in Figure 15 and Figure 16. Due to the enhanced nonlinearity of the inverse problem caused by the increased contrast, even in the absence of noise interference, conventional OPT-RMC-CC-CSI and OPT-DC-RMC-CC-CSI fail to accurately invert the target, and in fact, its reconstruction result has converged to a local optima. By comparing Figure 15b,c,e,f and Figure 16b,c,e,f, it is evident that using only the DC strategy at this stage cannot effectively improve the inversion results of higher-contrast targets under limited viewing angles. In contrast, both OPT-DC-WW_4_-RMC-CC-CSI and OPT-WW_4_-RMC-CC-CSI achieve accurate reconstructions by adapting WW_4_ that mitigates the nonlinearity of the inverse problem with a higher percentage of low-frequency data. We have plotted the corresponding PSNR curves in Figure 17. It demonstrates that under the situation of limited-aperture and dealing with higher-contrast targets, the inversion performance of the OPT-DC-WW_4_-RMC-CC-CSI combining DC and WW_4_ strategies surpasses all other types of optimal RMC-CC-CSI methods in terms of convergence speed, reconstruction accuracy and noise immunity. Furthermore, the results of DC-WW_4_-RMC-CC-CSI still remain close to that of OPT-DC-WW_4_-RMC-CC-CSI, which also validates the effectiveness and robustness of the BP image registration.

**Figure 18:**
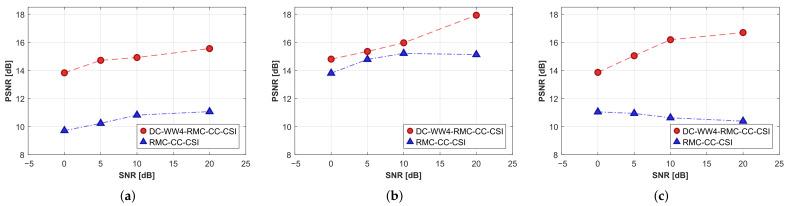
Performance degradation curves in terms of SNR levels when processing different datasets by RMC-CC-CSI and proposed method. (**a**) CrossRect. (**b**) SqTwoDisc. (**c**) SqTwoDisc with higher contrast.

The performance degradation curves of different methods for all datasets under different SNR levels are provided in Figure 18, which clearly presents the strength of the proposed algorithm. Analysis of all the above results demonstrates that only the DC-WW_4_ combined method achieves the most satisfactory reconstruction performance for a translational target. Notably, the WW_4_ strategy is also proved to be effective under limited-aperture scenario, exhibiting superior reconstruction accuracy compared especially in higher-contrast target configuration.

### 4.3. Complexity Analysis

**Figure 19:**
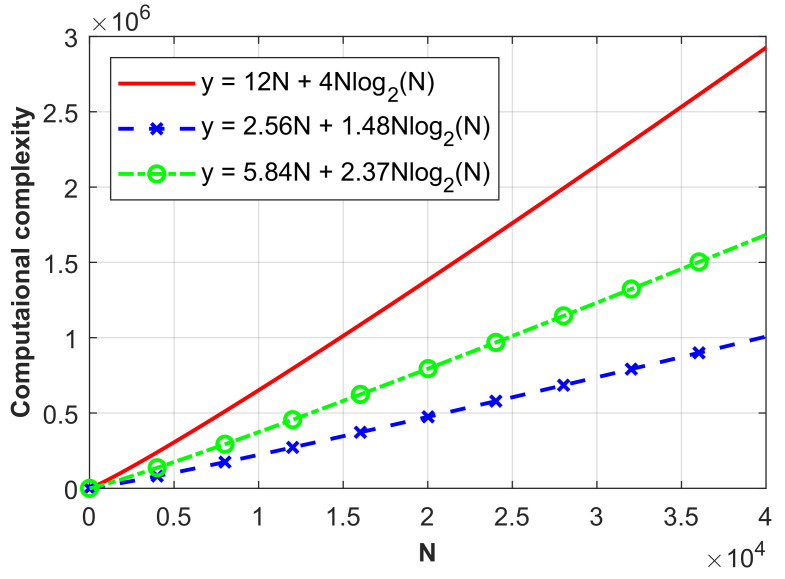
Comparison of computational complexity curves in terms of *N* when processing SqTwoDisc dataset using RMC-CC-CSI method and proposed method. $N_{f}$ = 1, *Q* = 1.

In the 2D case under the CC-CSI framework, the per-iteration computation complexity of this iterative method with single-incidence and single-frequency is $O(12N+4N\log_{2}N)$, which is given in Table I of [36]. Here, $N=N_{x}\times N_{y}$ denotes the dimension information of stiffness matrix $A\in C^{N\times N}$ defined according to the original grid size of the inversion domain $D={\mathrm{ROI}}_{\left(1\right)}$, which is divided with $N_{x}$ and $N_{y}$ grids in x− and y− directions. Under our DC-WW_*n*_-RMC-CC-CSI framework, with $\ell_{DC}=10\%\;\ell_{\max}$ and $\ell_{DC}=40\%\;\ell_{\max}$ representing low-noise and high-noise interference scenarios respectively, we conduct a specific complexity analysis on the SqTwoDisc dataset with SNR = 20 dB and 5 dB, for example. As shown in Figure 19 and Table 3, the proposed DC-WW_4_ exhibits significantly lower computational complexity than the original RMC-CC-CSI method.

### 4.4. Computational Performance

The implementation of RMC-CC-CSI requires the radar system to acquire additional translational motion data through different MIMO radar observation instants, inevitably increasing computational demands. To provide a more specific assessment, following the inversion parameters in Section 4.3, we executed $\ell_{\max}=5000$ iterations of both DC-WW_4_-RMC-CC-CSI and RMC-CC-CSI algorithms for the SqTwoDisc dataset on an AMD Ryzen AI 9 HX 370 laptop (Radeon 890 M 2.00 GHz CPU, 32 GB RAM), where the contraction interval of DC-WW_4_-RMC-CC-CSI is set at $\ell_{DC}=10\%\;\ell_{\max}$ and the total running time is $T_{tol}$. The average computational time comparison in Table 4 reveals DC-WW_4_-RMC-CC-CSI’s superior efficiency, with the original RMC-CC-CSI requiring obviously longer execution in each optimization iteration. Notably, the preliminary target positioning period involving BP imaging and registration contributes negligible time compared to the whole iterative optimization process.

## 5. Discussion

Based on all simulation results and analysis presented above, the following can be concluded:

1. Only the DC-WW_4_ combined method achieves the most satisfactory reconstruction performance for a translational target, exhibiting faster convergence speed and higher imaging accuracy.
2. DC-WW_4_-RMC-CC-CSI demonstrates better noise immunity than RMC-CC-CSI.
3. By introducing the DC strategy, DC-WW_4_-RMC-CC-CSI delivers better inversion performance with lighter cost of computational time compared to RMC-CC-CSI.
4. The WW_4_ strategy is also proved to be effective under limited-aperture scenario, exhibiting superior reconstruction accuracy and convergence speed, especially in higher-contrast target configuration. However, it is relatively sensitive to noise, with the overfitting phenomenon being particularly pronounced at low SNRs.

In summary, we have established the theoretical validity of the proposed DC-WW_*n*_ method and its advantages over baseline approaches through a set of simulation experiments under controlled conditions. However, several limitations must be acknowledged regarding its practical applicability:

1. The validation relies exclusively on synthetic data, which inherently assumes perfect knowledge of the background medium and idealized antenna responses. Real-world scenarios introduce challenges such as uncertain or heterogeneous background properties, imperfect and inconsistent antenna radiation patterns, and environmental clutter that deviates from simple additive white Gaussian noise models.
2. Only a single limited-aperture array geometry was tested in this work. It is usually recommended to perform similar simulation verification for specific geometries before actual deployment to evaluate performance boundaries. Therefore, our simulations are configured based on a practical scenario. However, real-world applications often require the design of different array geometries to meet specific requirements. These varied geometries will inevitably affect spatial diversity and the algorithm’s performance boundaries.

Although our inversion framework is general in theory, we cannot ignore the gap between simulation and measurement or exhaustively evaluate the algorithm’s performance across all possible geometries. Nevertheless, these limitations do not undermine the methodological contributions of this work. Rather, they define clear pathways for future research. In the future, we plan to focus on practical deployments through calibrated experimental testing with an actual system configuration. Such efforts will not only further validate the effectiveness of the proposed DC-WW_*n*_ strategy but also potentially extend its applicability to practical scenarios where model inaccuracy and data imperfections are prevailing challenges.

## 6. Conclusions

In this paper, a novel scheme aiming to solve the problem of efficient and accurate electromagnetic quantitative inversion of non-cooperative translational target in limited-aperture scenarios is proposed. By integrating a domain contraction (DC) strategy and the wavelength-dependent weighting (WW) of multi-frequency data to the original RMC-CC-CSI method, the efficacy of the proposed DC-WW_*n*_-RMC-CC-CSI method is validated using synthetic datasets. Systematic comparisons with different algorithms demonstrate three key advantages of DC-WW_*n*_ strategy: accelerated convergence, enhanced reconstruction accuracy, improved noise immunity and lighter computational burdens.

However, as the target’s contrast further increases, the limited-aperture inverse scattering problem can no longer be effectively solved within the framework of traditional iterative optimization algorithms. In such scenarios, neural network-based electromagnetic inversion algorithms will demonstrate their distinct advantages. In the future, building upon our relative motion-compensated inversion algorithm for translating targets, we will leverage deep learning to achieve intelligent electromagnetic inversion for complexly moving targets with even higher contrast.

## Figures and Tables

**Table 1:** The ideal center position matrix and ideal relative displacement matrix of different datasets under selected reference instant *c*.

Datasets	Matrix	Matrix Value: [m]
CrossRect	R	[−0.5, 0.8; −0.25, 0.7; 0, 0.6; 0.25, 0.5; 0.5, 0.4]^*T*^
	$R_{\Delta ,3}$	[−0.5, 0.2; −0.25, 0.1; 0, 0; 0.25, −0.1; 0.5, −0.2]^*T*^
SqTwoDisc	R	[−0.8, 0.9; −0.5, 0.6; −0.3, 0.7; 0, 0.75; 0.25, 0.5; 0.6, 0.3; 0.8, 0.4]^*T*^
	$R_{\Delta ,4}$	[−0.8, 0.15; −0.5, −0.15; −0.3, −0.05; 0, 0; 0.25, −0.25; 0.6, −0.45; 0.8, −0.35]^*T*^

**Table 2:** Estimated relative center position matrix of simulated datasets.

CrossRect ($\varepsilon_{r}$ = 1.5)		
**SNR [dB]**	**${\hat{R}}_{\Delta ,3}\left(1,:\right)$ (Upper) [m], ${\hat{R}}_{\Delta ,3}\left(2,:\right)$ (Lower) [m]**	**RMSE**
20	[−0.497, −0.247, 0, 0.245, 0.490]	0.0082
	[0.205, 0.105, 0, −0.106, −0.211]	
5	[−0.506, −0.248, 0, 0.248, 0.486]	0.0087
	[0.194, 0.102, 0, −0.107, −0.207]	
0	[−0.508, −0.246, 0, 0.262, 0.504]	0.0120
	[0.188, 0.102, 0, −0.113, −0.212]	
		
SqTwoDisc ($\varepsilon_{r1}$ = 1.5, $\varepsilon_{r2}$ = 2)		
**SNR [dB]**	**${\hat{R}}_{\Delta ,4}\left(1,:\right)$ (Upper) [m], ${\hat{R}}_{\Delta ,4}\left(2,:\right)$ (Lower) [m]**	**RMSE**
20	[−0.811, −0.504, −0.304, 0, 0.252, 0.606, 0.803]	0.0070
	[0.154, −0.148, −0.047, 0, −0.255, −0.455, −0.358]	
5	[−0.812, −0.510, −0.307, 0, 0.259, 0.592, 0.789]	0.0096
	[0.148, −0.153, −0.052, 0, −0.252, −0.454, −0.358]	
0	[−0.812, −0.510, −0.306, 0, 0.259, 0.610, 0.813]	0.0098
	[0.151, −0.151, −0.050, 0, −0.252, −0.454, −0.353]	
		
SqTwoDisc with higher contrast ($\varepsilon_{r1}$ = 3, $\varepsilon_{r2}$ = 6)		
**SNR [dB]**	**${\hat{R}}_{\Delta ,4}\left(1,:\right)$(Upper) [m], ${\hat{R}}_{\Delta ,4}\left(2,:\right)$(Lower) [m]**	**RMSE**
20	[−0.818, −0.509, −0.307, 0, 0.251, 0.602, 0.804]	0.0090
	[0.156, −0.148, −0.047, 0, −0.247, −0.447, −0.346]	
5	[−0.814, −0.509, −0.307, 0, 0.249, 0.604, 0.810]	0.0095
	[0.144, −0.146, −0.044, 0, −0.246, −0.445, −0.344]	

**Table 3:** Computational complexity of different iterative methods with single-incidence and single-frequency for processing the 2D SqTwoDisc dataset.

SNR	Method	$\ell_{\mathrm{DC}}$	ROI_(1)_	ROI_(2)_	Computational Complexity
20 dB	RMC-CC-CSI	$\ell_{\max}$	62 × 56 (N)	∖	$O(12N+4N\log_{2}N)$
	DC-WW_4_-RMC-CC-CSI	$10\%\;\ell_{\max}$	62 × 56 (N)	39 × 27 (≈30%N)	$O(2.56N+1.48N\log_{2}N)$
5 dB	RMC-CC-CSI	$\ell_{\max}$	62 × 56 (N)	∖	$O(12N+4N\log_{2}N)$
	DC-WW_4_-RMC-CC-CSI	$40\%\;\ell_{\max}$	62 × 56 (N)	41 × 27 (≈32%N)	$O(5.84N+2.37N\log_{2}N)$

**Table 4:** Running times of different methods for processing the 2D SqTwoDisc dataset. SNR = 20 dB, $\ell_{DC}=10\%\;\ell_{\max}$, $\ell_{\max}=5\times 10^{3}$.

Method	BP Imaging and Registration [s]	Total Time [s] ($T_{\mathrm{tol}}$)	Each Iteration [s] ($T_{\mathrm{tol}}/\ell_{\max}$)
RMC-CC-CSI	6.454	1580.7	0.3161
DC-WW_4_-RMC-CC-CSI	6.475	543.767	0.1078

## Data Availability

The data presented in this study are available on request from the corresponding author.

## Abbreviations

The following abbreviations are used in this manuscript:

DC	domain contraction
WW	wavelength-dependent weighting
RMC-CC-CSI	relative motion-compensated cross-correlated contrast source inversion
DC-WW	domain-contracted wavelength-dependent weighting
EM	electromagnetic
ISP	inverse scattering problem
BP	back-projection
BIM	Born iterative method
CSI	contrast source inversion
SOM	subspace-based optimization method
HMs	hybrid methods
ISAR	inverse synthetic aperture radar
CC-CSI	cross-correlated contrast source inversion
TM	transverse magnetic
FDFD	finite-difference frequency-domain
MIMO	multiple-input multiple-output
FDTD	finite-difference time-domain
PSNR	peak signal-to-noise ratio
ROI	region of interest
SNR	signal-to-noise ratio
